# Identification of a nucleotide in 5′ untranslated region contributing to virus replication and virulence of Coxsackievirus A16

**DOI:** 10.1038/srep20839

**Published:** 2016-02-10

**Authors:** Zhaolong Li, Xin Liu, Shaohua Wang, Jingliang Li, Min Hou, Guanchen Liu, Wenyan Zhang, Xiao-Fang Yu

**Affiliations:** 1 First Hospital of Jilin University, Institute of Virology and AIDS Research, Changchun, Jilin Province, China; 2 College of Life Science, Jilin University, Changchun, Jilin Province, China

## Abstract

Coxsackievirus A16 (CA16) and enterovirus 71 (EV71) are two main causative pathogens of hand, foot and mouth disease (HFMD). Unlike EV71, virulence determinants of CA16, particularly within 5′ untranslated region (5′UTR), have not been investigated until now. Here, a series of nucleotides present in 5′UTR of lethal but not in non-lethal CA16 strains were screened by aligning nucleotide sequences of lethal circulating Changchun CA16 and the prototype G10 as well as non-lethal SHZH05 strains. A representative infectious clone based on a lethal Changchun024 sequence and infectious mutants with various nucleotide alterations in 5′UTR were constructed and further investigated by assessing virus replication *in vitro* and virulence in neonatal mice. Compared to the lethal infectious clone, the M2 mutant with a change from cytosine to uracil at nucleotide 104 showed weaker virulence and lower replication capacity. The predicted secondary structure of the 5′UTR of CA16 RNA showed that M2 mutant located between the cloverleaf and stem-loop II, affected interactions between the 5′UTR and the heterogeneous nuclear ribonucleoprotein K (hnRNP K) and A1 (hnRNP A1) that are important for translational activity. Thus, our research determined a virulence-associated site in the 5′UTR of CA16, providing a crucial molecular target for antiviral drug development.

Coxsackievirus A16 (CA16) and enterovirus 71 (EV71) are two main causative pathogens of hand, foot and mouth disease (HFMD). Both viruses belong to the *Enterovirus* genus of the *Picornaviridae* family and possess a single positive-stranded RNA virus with an icosahedral symmetry structure. The RNA genome of enteroviruses consists of three types of regions: non-coding regions including the 5′ untranslated region (5′UTR) and 3′ untranslated region (3′UTR); a structural region P1 including VP1, VP2, VP3 and VP4; and non-structural regions P2 and P3 including 2A, 2B, 2C, 3A, 3B, 3C and 3D^1^.

Upon entering a permissive host cell, the enterovirus genome first serves as a template for translation, producing the viral polyprotein, and then becomes a template for replication of the minus strand. The 5′UTR is fundamentally required for these functions by interacting with cellular proteins^2^,^3^,^4^,^5^. The 5′UTR RNA of picornavirus contains two secondary structures. Viral RNA replication is closely related to the 5′-terminal cloverleaf structure, whereas the initiation of translation on the 5′UTR involves an internal ribosome entry sites (IRESs) that occupy most of the rest of the viral 5′UTR^6^,^7^. Several RNA binding proteins, such as heterogeneous nuclear ribonucleoprotein (hnRNP A1) are involved in the translational control of mRNAs containing IRESs. These hnRNP proteins constitute IRES tran-acting factors (ITAFs) that modulate the activity of IRES sequences present in the 5′UTR of viral or cellular mRNAs^8^. Poliovirus, EV71 and bovine enterovirus all require eIF2, eIF3, eIF4A, eIF4G, eIF4B, eIF1A, and a single ITAF, poly(C) binding protein 2 (PCBP2) which represent a common set of canonical initiation factors and ITAFs for the formation of an efficient 48S complex *in vitro* for reconstitution of initiation^8^.

In a previous report, the 5′UTR region in EV71 was shown to be associated with virus replication via interaction with poly(C)-binding protein 1 (PCBP1)^9^. hnRNP A1 not only is an ITAF that binds specifically to the 5′UTR of EV71 and regulates IRES-dependent translation but it also binds to the 5′UTR of Sindbis virus (SV) to facilitate translation^10^. A single nucleotide change from cytosine to uracil at base 158 of the 5′UTR was found to reduce viral translation and virulence of EV71 in mice^9^ and was associated with a fatal clinical case^11^. SL II in the 5′UTR of coxsackievirus (CV) B1 and B3 determines their cardiovirulence phenotype^12^,^13^. For CA16 viruses, a neonatal mouse model for vaccine evaluation was established, and inactivated or recombinant virus-like particle vaccines have been developed and evaluated^13^,^14^,^15^,^16^. The pathogenic mechanism of CA16 infection which induces the apoptosis of neural or non-neural cells also has been investigated^16^,^17^. However, the mechanism of CA16 virus replication has not been well-studied, and the function of its 5′UTR during viral infection is unknown until now.

In this study, we determined for the first time that the 5′UTR of CA16 is crucial for virus replication and its virulence in a neonatal mouse model. We identified cytosine at base 104 located between the cloverleaf and stem-loop II of the 5′UTR of CA16 as a virulence determinant, which decreased the viral RNA replication, and consequently affected the clinical score and mortality in the neonatal mouse challenge model. Further investigations showed that the virulence-associated base 104 in the 5′UTR of CA16 RNA also affected translational activity mediated by the IRES that occupies most of the rest of the 5′UTR through abrogating its interaction with cellular proteins hnRNP K and hnRNP A1 proteins. Taken together, our findings indicate that the 5′UTR of CA16 plays an important role in CA16 viral replication, as well as viral RNA synthesis and translational activity.

## Results

### Effect of various CA16 strains on disease induction and survival rate in neonatal mice

**Figure 1:**
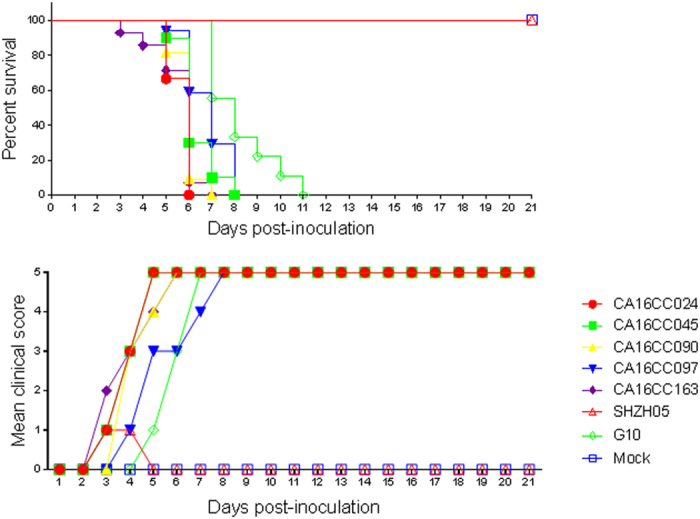
Assessment of morbidity and mortality in newborn mice infected with circulating CA16 CC024, CC045, CC090, CC097, CC163, the prototype G10 and SHZH05 viruses. One-day-old ICR mice were intracerebrally inoculated with different virus strains at 10^4.5^ CCID50 ml^−1^ or MEM cell culture medium. Survival rates and clinical scores were then monitored and recorded daily after infection. Control mice were given cell culture medium instead of virus suspension. Each group contained 8–10 mice, and results were obtained from three independent experiments. Varying grades of clinical disease were identified as: 0, healthy; 1, lethargy and inactivity; 2, wasting; 3, limb shaking and weakness; 4, hind-limb paralysis; 5, moribund or dead.

The lethality of a series of CA16 strains, including circulating CA16 CC strains, the prototype G10 and SHZH05 strain, was analyzed at the dosage of 10^4.5^ CCID_50_ ml^−1^ in neonatal mice. The SHZH05 strain was found to be different from circulating CA16 CC strains (CC024, CC045, CC090, CC097 and CC163) isolated from patients in Changchun and the G10 strain. Mice injected with SHZH05 presented low clinical scores and 100% survival rate, while those injected with circulating CA16 CC and G10 strains displayed high clinical scores and 0% survival rates, dying between days 3 to 11 after injection (Fig. 1). These results suggest that some sites in the genomic sequence of the SHZH05 strain compared with other lethal strains induced these different outcomes. Finding virulence-associated sites may help us to develop novel therapeutic targets against HFMD.

### Alignment of 5′UTR sequences of various CA16 strains and construction of infectious clones

**Figure 2:**
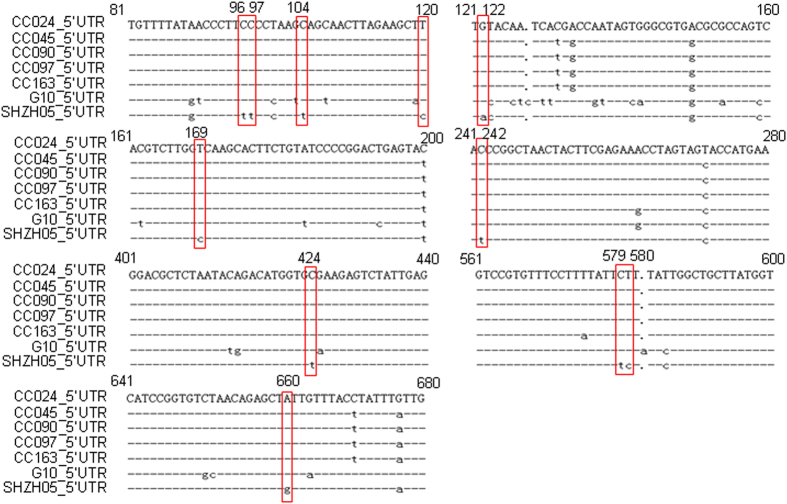
Identification of nucleotides located in 5′UTR of lethal CA16 strains (CC024, CC045, CC090, CC097, CC163) and the prototype G10 strain but not in the non-lethal SHZH05 strain.

**Figure 3:**
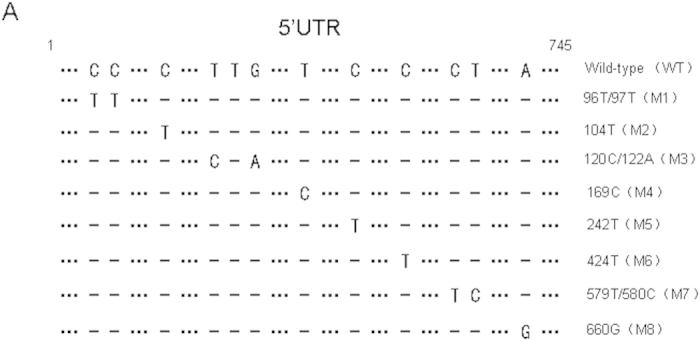
Construction of infectious clones of WT and various mutants with nucleotide alterations. The full-length genome of CA16 CC024 was inserted into pBlueScript SK (+) between the T7 promoter and poly A tail. Alignment of 5′UTR sequences of infectious clones of WT and mutant (M1–M8) viruses.

The 5′UTR region in EV71, which is another main causative pathogen of HFMD, has been associated with virus replication and virulence^18^,^19^,^20^. In order to determine whether the 5′UTR region of CA16 also plays a key role in viral virulence, 5′UTR sequences of five lethal CA16 CC strains and the prototype G10 as well as non-lethal SHZH05 strain were aligned. We speculated that certain sites, which are conserved nucleotides among CA16 CC and G10 strains but different from that in the SHZH05 strain, may correlate with pathological changes and mortality of neonatal mice (Fig. 2). In order to test the above assumptions, an infectious cDNA copy of the CC024 genome was cloned based on the representative lethal CC024 strain sequence and designated as the wild-type (WT).The full-length genome of the CA16 CC024 strain was constructed and inserted into the pBlueScript SK (+) vector between the T7 promoter and poly A tail. Mutant infectious clones with nucleotides located in the 5′UTR region of SHZH05 that are different from CC024, designated M1 to M8, were obtained by site-directed mutagenesis and confirmed by sequencing (Fig. 3).

### 5′UTR of CA16 affects lethality and virus replication in infected neonatal mice

**Figure 4:**
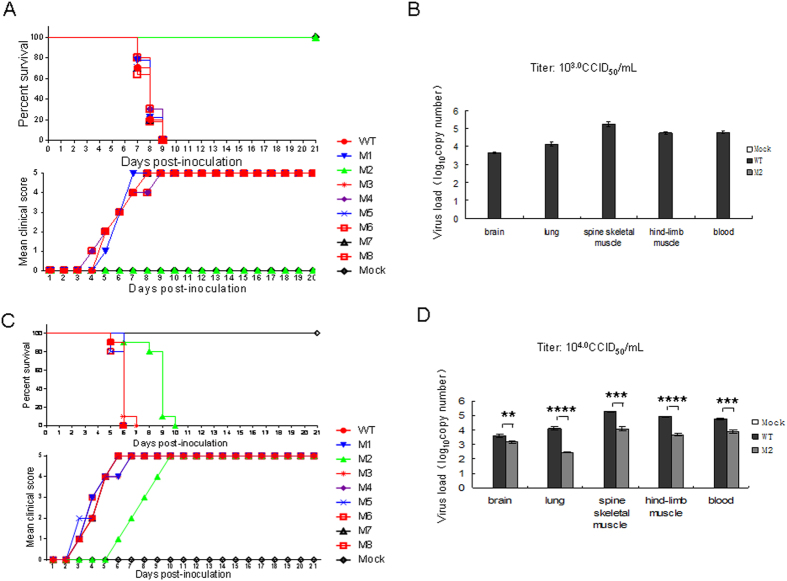
Correlation of 5′UTR nucleotides with viral virulence of CA16 in neonatal mice. (**A,C**) Viruses from HEK293T cells transfected with WT or various mutant infectious clones (10 μL) were intracerebrally inoculated into neonatal mice at 10^3.0^ CCID_50_ ml^−1^ (**A**) or 10^4.0^ CCID_50_ ml^−1^ (**C**), and survival rates and clinical scores were monitored and recorded daily after infection. Mock-infected mice were given culture medium from cells transfected with negative empty vector. (**B,D**) Viral load variations in various tissues of mice inoculated with WT or M2 viruses at 10^3.0^ CCID_50_ ml^−1^ (**B**) or 10^4.0^ CCID_50_ ml^−1^ (**D**). Virus loads were assessed by RT-qPCR with primers specific for the GAPDH or for CA16 VP1 RNA in samples of the brain, lung, spine skeletal muscle, hind-limb muscle and blood from infected mice. GAPDH was used as a control. The results represent the mean virus loads [log_10_ copies (mg tissue)^−1^ or log_10_ copies (ml blood)^−1^] ± SD (three mice per group, repeated three times). ^*^P < 0.05, ^**^P < 0.01, ^***^P < 0.001, ^****^P < 0.0001.

To investigate potential differences in survival rates in neonatal mice, an empty vector as negative control, WT infectious clone or various mutant infectious clones plus pcDNA3.1-T7 DNA Pol were transfected into human embryonic kidney 293T (HEK293T) cells. At 72 h post-transfection, cells and supernatants of WT and mutant viruses from transfected HEK293T cells were harvested together, frozen in −80 °C and thawed several times to release viruses, respectively. The samples were then centrifuged at 4000 rpm for 5 min to remove cellular debris and filtered through a 0.2-μm pore size membrane before titering in Vero cells. Subsequently, the viruses were injected intracerebrally into 1-day-old neonatal mice at the dosage of 10^3.0^ CCID50 ml^−1^ or 10^4.0^ CCID50 ml^−1^. A sample prepared from cells transfected with a control vector was used as the negative control. Clinical scores and the mortality rate of newborn mice infected with viruses were examined. We found mutant M2 viruses with a nucleotide change from cytosine to uracil at base 104 did not induce any elevation in clinical score or death in neonatal mice at the dosage of 10^3.0^ CCID50 ml^−1^. Meanwhile, WT, M1 and M3–M8 mutants all resulted in different clinical scores, which increased between grade 1 to 5 from day 4 or 8 and caused 100% mortality by day 9 after injection in neonatal mice (Fig. 4A). Mice that were infected with mutant M2 virus at the 10^4.0^ CCID_50_ ml^−1^ dosage displayed clinical symptoms on day 5 post-infection and had a mean clinical score of grade 5 and mortality of 100% by day 10 (Fig. 4C). However, the mice injected with WT, M1 or M3–M8 viruses began to show clinical score on day 2 after the injection and had a mean clinical score of grade 5 and mortality of 100% by day 7 (Fig. 4C). Mice infected with the M2 virus showed delayed clinical score compared with those infected with WT, M1 or M3-M8 mutant viruses at the dosage of 10^4.0^ CCID_50_ ml^−1^. As expected, mice that were injected with negative control alone showed a clinical score of grade 0 and had a 100% survival rate.

To further understand the correlation between the pathological changes and the replication of CA16 viruses in infected mice, viral loads were measured in various tissues including brain, lung, spine skeletal muscle, hind-limb muscle and blood from WT- or M2-infected mice by reverse transcription quantitative real-time PCR (RT-qPCR). When the mice infected with WT virus started to display a clinical score of grade 4 by presenting hind-limb paralysis at day 5 (10^4.0^ CCID_50_ ml^−1^) or day 7 (10^3.0^ CCID_50_ ml^−1^) after injection, mice were taken from both the WT and M2 groups for analysis of virus load. The results showed almost no virus replication in mice infected with M2 at the dosage of 10^3.0^ CCID_50_ ml^−1^ compared with those infected with WT (Fig. 4B). Viral loads in various tissues infected with M2 viruses were lower than those infected with WT at the dosage of 10^4.0^ CCID_50_ ml^−1^ (Fig. 4D). These results suggest that the nucleotide cytosine at base 104 in the 5′UTR affected the replication of CA16 virus in neonatal mice.

### 5′UTR of CA16 affects pathological changes in infected neonatal mice

**Figure 5:**
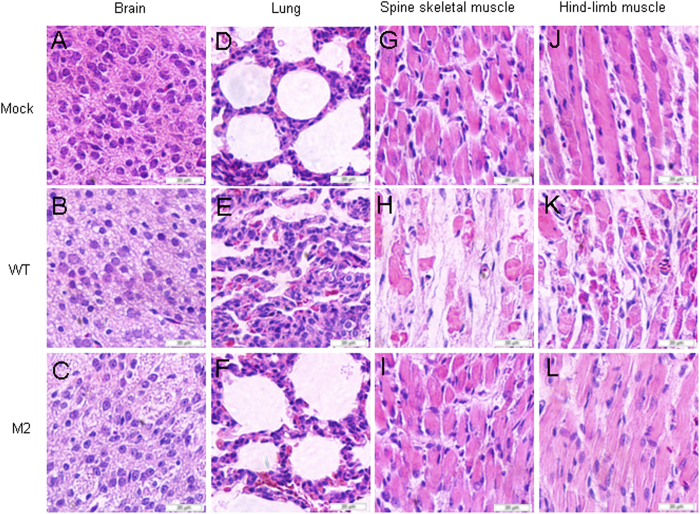
Pathological analysis of WT or M2 infected neonatal mice. One-day-old ICR mice were intracerebrally inoculated with medium or WT or M2 virus from transfected HEK293T cells at 10^4^ CCID_50_ ml^−1^. No histological change was observed in the brain of the non-infected medium control (**A**) or WT (**B**) or M2 (**C**) infected mice. No histological change was observed in the lung (**D,F**) or hind limb muscle (**G,I**) or spinal muscle (**J,L**) of the non-infected medium control or M2 infected mice. Mice infected with WT viruses exhibited severe alveolar shrinkage (**E**) in the lung tissue. Mice infected with WT viruses (grades 4 to 5) exhibited signs of severe necrosis, including muscle bundle fracture, dissolution of muscle fiber cells, nuclei shrinkage and swelling (H and K). A to L, magnification 400× .

The CA16CC024 strain has been shown to cause severe lesions in the lung, spine skeletal muscle and hind-limb muscle. To understand whether mutant M2 viruses would affect pathological changes resulting in death in infected neonatal mice, we performed a systematic pathological analysis of various tissues, including brain, lung, spine skeletal muscle and hind-limb muscle, from infected mice with a grade 5 clinical score that were challenged with WT viruses at 10^4^ CCID_50_ ml^−1^. We observed that no pathological changes in the brain of non-infected mice or mice infected with WT or M2 virus (Fig. 5A–C). Spine skeletal muscle and hind-limb muscle fibers in non-infected mice (Fig. 2G,J) and infected with M2 virus mice (Fig. 2I,L) did not exhibit signs of severe necrosis, including muscle bundle fracture, dissolution of muscle fiber cells, and swelling and shrinkage of the nuclei, when compared with mice infected with the WT virus (Fig. 2H,K). The M2 virus (Fig. 2F) did not cause obvious lung tissue lesion or severe alveolar shrinkage when compared with effects of the WT virus (Fig. 2E). In the negative control group, no pathological changes were found in the lung (Fig.2D). These results demonstrated that the M2 mutant with the alteration in the 5′UTR of CA16 affected its virulence and attenuated lesions in the lung, spine skeletal muscle and hind-limb muscle.

### 5′UTR of CA16 affects virus replication *in vitro*

**Figure 6:**
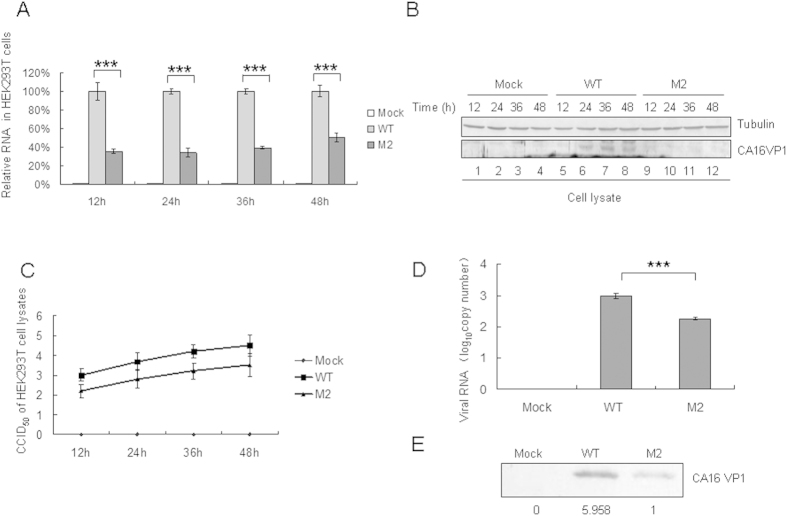
Effect of WT or M2 mutant 5′UTR of CA16 on viral RNA synthesis and viral translational capacity in transfected HEK293T cells. HEK293T cells were transfected with same amount of the WT or M2 infectious clone or empty vector, and were harvested at 12 h, 24 h, 36 h and 48 h after transfection. (**A**) RNA was extracted from a portion of each sample, treated with Dnase to degrade the transfected plasmid. DNA and then analyzed by RT-qPCR with primers specific for GAPDH or CA16 VP1 RNA. GAPDH was used as a control. The RNA level obtained by transfection with WT 5′UTR was normalized to 100%. (**B**) A portion of each cell sample was used to detect the VP1 protein by Western blot. (**C**) A portion of each cell sample was used to detect the viral titer. (**D**) Viral loads in supernatant of transfected HEK293T cells at 48h after transfection were detected by RT-qPCR with primers specific for CA16 VP1 RNA. (**E**) The VP1 protein of the WT or M2 virus in the supernatant was detected by Western blot using VP1 antibody. ^*^P < 0.05, ^**^P < 0.01, ^***^P < 0.001, ^****^P < 0.0001.

To confirm whether the 5′UTR of CA16 affects aspects of virus replication including viral RNA synthesis and translational *in vitro*, levels of viral RNA and proteins were examined in HEK293T cells transfected with pcDNA3.1-T7 DNA Pol and equivalent amounts of the WT or mutant M2 infectious expression vector. The transfected cells were harvested at 12 h, 24 h, 36 h and 48 h later. Detection of viral RNA levels by RT-qPCR showed that M2 replicated at a lower level than the WT in HEK293T cells (Fig. 6A). The VP1 expression detectd by Western blot in HEK293T cells transfected with WT infectious expression vector was obviously greater than that in cells transfected with the M2 mutant at 24 h, 36 h and 48 h (Fig.6B lanes 6, 7, 8 and 10, 11, 12). The viral titer in HEK293T cells transfected with the WT infectious expression vector was obviously higher than that in cells transfected with the M2 mutant at any time point (Fig. 6C). Levels of viral RNA and VP1 protein of the WT strain in the supernatant were found to be higher than those of the M2 mutant by RT-qPCR and Western blot analysis, respectively (Fig. 6D,E).

**Figure 7:**
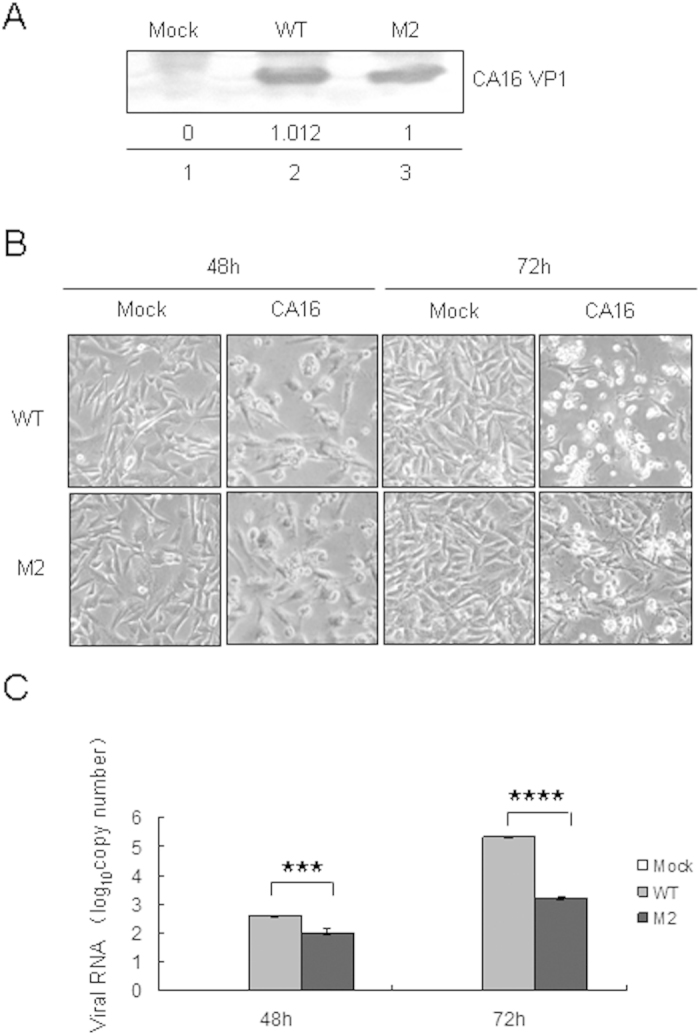
Effect of WT or M2 mutant 5′UTR of CA16 on viral RNA synthesis and viral translational capacity in infected RD cells. (**A**) Equal amounts WT and M2 viruses were determined using a VP1 antibody by immunoblotting analysis. (**B**) CPEs in RD cells induced by equal amounts of WT and M2 viruses were imaged via light microscopy at 48 h and 72 h after transfection. (**C**) Viral loads in the supernatant of RD cells were detected by RT-qPCR. The results represent at least three independent experiments. ^*^P < 0.05, ^**^P < 0.01, ^***^P < 0.001, ^****^P < 0.0001.

To further confirm the results above, RD cells were inoculated with the same amount of WT or M2 virus, as determined by Western blot analysis of the VPI protein (Fig. 7A). Stronger cytopathic effects (CPEs) were observed in RD cells infected with the WT than in those with the M2 virus, especially at 72 h (Fig. 7B). Supernatants of RD cells were then harvested at 48 h and 72 h and used to examine viral RNA levels by RT-qPCR (Fig. 7C). The analysis suggested that the WT virus replicated more rapidly than M2 virus in RD cells. These results showed that the M2 mutant with a nucleotide change from cytosine to uracil at base 104 in the 5′UTR had a lower replication capacity than WT both in RD and HEK293T cell lines.

### 5′UTR of CA16 affects translational activity *in vitro*

**Figure 8:**
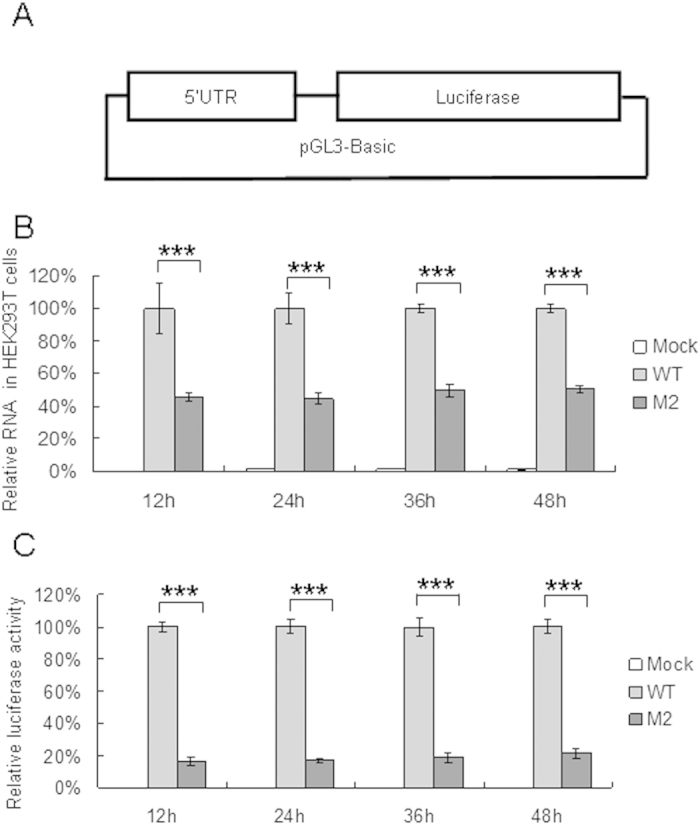
Effect of nucleotide substitution on RNA replication and translational capacity mediated by CA16 5′UTR. (**A**) Construction of reporter plasmid to determine translational activity mediated by CA16 5′UTR. (**B**) Effect of base 104 of 5′UTR on RNA replication in cells. WT or M2 vector expressing luciferase were transfected into HEK293T cells, and cells were harvested at 12 h, 24 h, 36 h and 48 h after transfection. RNA was extracted from a portion of each cell sample and analyzed by RT-qPCR with primers specific for GAPDH RNA or CA16 5′UTR RNA. GAPDH was used as a control. The RNA level obtained from transfection with WT 5′UTR was normalized to 100%. (**C**) Effect of base 104 of 5′UTR on translational activity in cells. Luciferase activiy was detected in a portion of each cell sample.and the viral replication rate was expressed as a fold increase in luciferase activity. ^*^P < 0.05, ^**^P < 0.01, ^***^P < 0.001, ^****^P < 0.0001.

The 5′UTR of WT and M2 strains were inserted into the pGL3-Basic vector (Fig. 8A) and used to perform luciferase assays in HEK293T cells. HEK293T cells were transfected with VR1012, pGL3-WT or pGL3-M2 expression vector. The cells were harvested at 12 h, 24 h, 36 h and 48 h later. Detection of viral RNA levels by RT-qPCR showed that M2 replicated at a lower level than the WT in HEK293T cells (Fig. 8B). Luciferase activeties showed that the nucleotide change from cytosine to uracil at base 104 also reduced translational activity mediated by the 5′UTR of the M2 mutant (Fig. 8C).

### 5′UTR from lethal strain but not M2 mutant with uracil at base 104 of CA16 RNA interacts with hnRNP K and hnRNP A1 proteins

**Figure 9:**
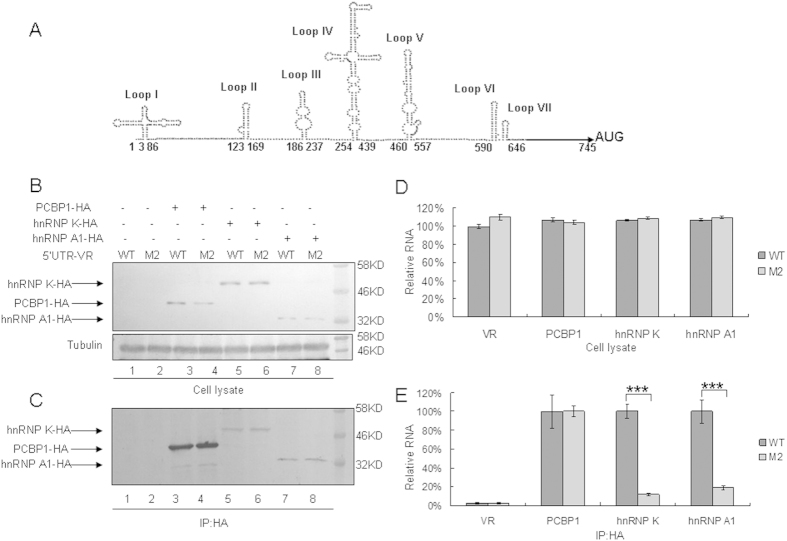
Interactions of WT or M2 mutant 5′UTR of CA16 with cellular proteins hnRNP K, hnRNP A1 and PCBP1. (**A**) Structural prediction of CA16 5′UTR using Mfold software. The nucleotide change C104T is located at the linker between loop I and II. (**B**) Expression of cellular proteins after transfection with 5′UTR of CA16. VR1012, hnRNP K-HA, hnRNP A1-HA or PCBP1-HA was co-transfected with WT 5′UTR or M2 mutant expression vector into HEK293T cells. Cell lysate were prepared at 48 h after transfection. Part of each cell lysate was dissolved in 1 × loading buffer for immunoblotting analysis. (**C**) Immunoprecipitation assay. Most of each cell lysates was incubated with anti-HA agarose beads at 4 °C for 3 h. Following washing and dissociation, part of each bead pellet was re-suspended in 1 × loading buffer for immunoblotting analysis, and another part was used for RNA extraction. (**D**) RNA levels of CA16 5′UTR in cell lysates. RNA was extracted from a portion of each set of transfected HEK293T cells and then analyzed by RT-qPCR using primers specific for GAPDH RNA or CA16 5′UTR RNA. GAPDH was used as a control. The RNA level in the presence of VR1012 and WT 5′UTR was normalized to 100%. (**E**) Interaction of various cellular proteins with CA16 5′UTR. RNA extract from immunoprecipitation was subjected to RT-qPCR analysis with primers specific for CA16 5′UTR RNA. The RNA level in the presence of PCBP1, hnRNP K or hnRNP A1 and WT 5′UTR was normalized to 100%. The RNA level in the presence of PCBP1 and WT 5′UTR was normalized to 100% in the VR1012 negative control group. Errors bars represent the SD from triplicate wells within one experiment. Results represent at least three independent experiments. ^*^P < 0.05, ^**^P < 0.01, ^***^P < 0.001, ^****^P < 0.0001.

Cellular proteins important for virus replication, such as hnRNP K, hnRNP A1 as well as PCBP1, have been shown to interact with the EV71 5′UTR^9^,^21^,^22^. The secondary structure of the 5′ UTR of CA16 RNA predicted by Mfold (Fig. 9A) showed that base 104 in the 5′ UTR is located at the linker between the coverleaf and stem-loop II. While the linker in the 5′ UTR of EV71 also has been demonstrated to be important for binding with cellular proteins^9^,^23^. This model implies that the nucleotide cytosine at base 104 in the 5′UTR is a major determinant for viral translation and CA16 virulence. Subsequently, we detected whether the 5′UTR of CA16 RNA interacts with these proteins by using an immunoprecipitation assay and compared the binding ability of WT 5′UTR and M2 mutant 5′UTR to interact with these proteins. VR1012, hnRNP K-HA, hnRNP A1-HA or PCBP1-HA was co-transfected with the WT 5′UTR or M2 mutant 5′UTR expression vector into HEK293T cells. An anti-HA antibody conjugated to agarose beads was used to immunoprecipitate HA-tagged proteins from lysates of transfected HEK293T cells. As expected, hnRNP K-HA, hnRNP A1-HA and PCBP1-HA were expressed in 293T cells (Fig. 9B) and could be immunoprecipitated from cell lysates (Fig. 9C). 5′UTR RNA levels in cell lysates from the WT 5′UTR or M2 mutant group were similar in the presence of any of the tested proteins, hnRNP K, hnRNP A1 or PCBP1 (Fig. 9D). However, we found that the M2 mutant with the base 104 alteration in its 5′UTR showed profoundly reduced hnRNP K and hnRNP A1 binding compared to the WT 5′UTR (Fig. 9E). Both the WT 5′UTR and M2 mutant 5′UTR maintained the same ability to interact with the PCBP1 protein (Fig. 9E). No RNA was detected in the VR1012 group, showing that the interactions between viral RNA and these proteins were specific (Fig. 9E).

## Discussion

In this study, we identified that the C-to-T exchange at nucleotide 104 between the cloverleaf and the stem-loop II in the 5′UTR of CA16 resulted in lower viral RNA replication and translational activity in neonatal mice and at the cellular level *in vitro*. The 5′UTR of enteroviruses, including poliovirus, CVB3 and EV71, is important for viral RNA and protein syntheses. The proposed secondary structure of the 5′UTR of EV71 or CVB3 contains seven predicted structural domains (I to VII)^9^,^23^. Domain I, a cloverleaf-like structure formed by the 5′-terminus of the RNA (approximately 90 nt), is generally agreed to regulate synthesis of the plus-strand RNA^24^,^25^, which also contributes to the initiation of translation^21^. Domains II to VI house the IRES element^22^, although the minimal IRES requires only domains II, IV and V^26^,^27^.

Using the neonatal mice challenge model, we found that a series of CA16 Changchun strains and the prototype G10 but not the SHZH05 strain could induce more severe clinical scores and death (Fig. 1). We also determined that the mutant M2 infectious clone containing a nucleotide change from cytosine to uracil at base 104 located between the cloverleaf and stem-loop II could not induce pathological changes and death in neonatal mice. Shiroki *et al*. reported on the generation of mutants of the virulent Mahoney strain of poliovirus by disruption of nucleotides 128 to 134 at stem-loop II within the 5′UTR. Four of these mutants replicated well in human HeLa cells but poorly in mouse TgSVA cells that had been established from the kidney of the poliovirus-sensitive transgenic mouse^28^. Further study determined that a mutation at nucleotide 107, specifically a change from uracil to adenine, resulted in a recovery of IRES activity in a cell-free translation system from TgSVA cells and a return to a neurovirulent phenotype similar to that of the Mahoney strain in mice^29^. Those authors also reported that SLII-2 was defective in genomic RNA synthesis and viral protein synthesis^30^. Nucleotide 104 in the 5′UTR of CA16 that we identified as the viral determinant is very close to a single conserved nucleotide 102 identified by Nidia *et al*. which locates between the cloverleaf and IRES in the 5′UTR of poliovirus and is responsible for the observed change of the neurovirulence phenotype^31^.

The M2 virus with nucleotide cytosine to uracil at base 104 in the 5′UTR of CA16 was found not only to be important for viral virulence of CA16 in neonatal mice, but is also resulted in lower levels of replication as evidenced by lower viral RNA synthesis and viral protein expression, in both HEK293T and RD cell lines (Figs 6 and 7). The 5′UTR luciferase assay further showed that the M2 5′UTR induced weaker luciferase expression than that by the WT 5′UTR (Fig. 8). These results confirmed that the nucleotide substitution in the M2 affected virus replication through modulating the RNA level and translational activity for virus replication *in vitro*. The virus replication involves the cloverleaf, whereas initiation of transcription on the 5′UTR is mediated by and the IRES that occupies most of the rest of the 5′UTR. However an overlapping zone within the 5′UTR may be present which would influence both processes. Initiation on Type 1 IRESs of viruses, including poliovirus, EV71 and BEV all require cellular proteins with the cloverleaf^8^.

Recent studies associated the 5′UTR of EV71 with virus replication via binding cellular proteins including PCBP1, hnRNP A1 and hnRNP K^18^,^20^,^32^,^33^, which are invoved with virus replication and translation. The proposed model predicted by Mfold software showed that nucleotide 104 in the M2 virus is located between the cloverleaf and stem loop II (Fig. 9A). Our data confirmed that the M2 5′UTR could not interact with hnRNP K or hnRNP A1 (Fig. 9).

In summary, we conclude that the 5′UTR of CA16 is important for virus replication, and the nucleotide cytosine at base 104 within this region is a viral determinant. However, other sites that differentiate between the SHZH05 strain and CA16 CC strains which affect the virulence in neonatal mice may exist since the M2 virus was still observed to be more virulent than the SHZH05 virus. Our study provides new insight into the pathogenesis of CA16-related diseases and potentially facilitate the development of a small molecular inhibitor based on the newly identified the region between the cloverleaf and stem loop II in the M2 virus that can be used to inhibit virus replication.

## Materials and Methods

### Plasmid construction

RNA of CA16 CC024 was extracted from the supernatant of virus-infected Vero cells using Trizol (Invitrogen, Carlsbad, CA, USA) and reverse transcribed using oligo (dT) primers and M-MLV reverse transcriptase (Invitrogen) according to the manufacturer’s instructions. The resulting cDNA was used for amplification of three CA16 fragments with primers P1-P6 as shown in Table 1. The PCR products were sub-cloned into the *Not*I/*Sac*I sites of pBlueScript SK(+). pcDNA3.1-T7 DNA Pol was a gift from CL Jiang (College of Life Science, Jilin University). Mutants derived from the CA16 infectious WT clone was constructed using PrimeSTAR HS DNA Polymerase (Takara, Shiga, Japan) and primers P7-P22 as shown in Table 1 by PCR-based mutagenesis. All infectious clones were confirmed by sequencing the whole genome. The 5′UTR-luciferase plasmid was constructed as follows. 5′UTR of CA16 (WT or M2) was amplified using primers P23-P26 (Table 1), and the resulting product was inserted into the *Xho*I/*Hin*dIII sites of pGL3-Basic (Promega, Madison, WI, USA). PCBP1-HA, hnRNPK-HA and hnRNPA1-HA were amplified from cDNAs of RD cells with primers P27–P32 (Table 1) and inserted into VR1012. 5′UTRs of WT and M2 of CA16 were amplified from infectious clones of the WT and M2 mutant with primers P33 and P34 (Table 1), and then the 5′UTRs were inserted into VR1012. VR1012 was used is a negative control vector which was described in our previous study^30^.

### Cells culture, transfection and viruses

Human embryonic kidney 293T cells (HEK293T, no. CRL-11268), Human rhabdomyosarcoma RD cells (no. CCL-136) and African green monkey kidney Vero cells (no. CCL-81) were obtained from American Type Culture Collection (Manassas, VA, USA) and cultured as a monolayer in Dulbecco’s modified Eagle’s medium and minimum essential medium (Hyclone, Logan, UT, USA) supplemented with 10% heat-inactivated (56 °C, 30 min) fetal calf serum (FCS, GIBCO BRL, Grand Island, NY, USA) and maintained at 37 °C with 5% CO_2_ in a humidified atmosphere. A 70% confluent monolayer of HEK293T cells in a T25 flask (BD Biosciences, Franklin Lakes, NJ, USA) was transfected with pcDNA3.1-T7 DNA Pol (1 μg) and CA16 infectious clone (2 μg) or mutants digested by the restriction enzyme *Sac* I (Takara) using 9 μl Lipofectamine 2000 reagent (Invitrogen) and then incubated at 37 °C in 5 ml of DMEM (10% FCS). The cells were washed, and the medium was replaced with DMEM (10% FCS) at 6 h post-transfection. At 72 h after post-transfection, cells and supernatants of WT and mutant viruses from transfected HEK293T cells were harvested together, frozen at −80 °C and thawed several times to release the viruses. The samples were then centrifuged at 4000 rpm for 5 min to remove cellular debris and filtered through a 0.2-μm pore size membrane.

GenBank/EMBL/DDBJ accession numbers for coxsackievirus A16 strains CC024, CC045, CC090, CC097, CC163, G10 and SHZH05 are KF055238, KF055241, KF055243, KF055244, KF055245, U05876 and EU262658, respectively.

CA16 CC024 and mutants viruses were propagated using Vero cells as previously described^34^,^35^. Briefly, cells were grown to 80% confluence in a T75 flask, washed twice with phosphate-buffered saline (PBS) and incubated with virus at 37 °C for 1 h. During adsorption, the flask was gently agitated at 15-min intervals. Following adsorption, the virus-containing medium was replaced with fresh medium containing 2% FCS, followed by incubation at 37 °C in 5% CO_2_. Once 90% of the cells showed CPEs, the viral supernatant was harvested and centrifuged at 4,000 rpm for 5 min. The clear supernatant was then transferred to a new tube and stored at −80 °C.

### Virus titer

The virus titer was determined by measuring the 50% tissue culture infective dose (CCID_50_) in a microtitration assay, as described previously^36^. Briefly, Vero cells were seeded into 96-well plates and incubated at 37 °C for 24 h. Virus-containing supernatant was serially diluted 10-fold, and 100 μl was added per well in triplicate. The cytopathic effects (CPEs) were observed once per day until the experimental endpoint. The viral titer was determined in Vero cells according to the Reed-Muench method (Reed and Muench, 1938).

### RNA extraction and RT-qPCR

For RT-qPCR, viral RNA was extracted using TRIzol from equal volumes of cells or supernatants of HEK293T cells transfected with infectious clones (WT or mutant strains) or RD cells infected with WT or mutant viruses or equal amounts (by weight) of fresh homogenates from tissues of mice injected with viruses. Viral RNA samples from cells transfected with infectious clones, were treated with DNase by incubation in 10 μL of diethyl pyrocarbonate (DEPC)-treated water with 1 × RQ1 RNase-free DNase Buffer, 1 μL RQ1 RNase-free DNase (Promega) and 4 U RNase inhibitor (New England BioLabs, Ipswich, MA, USA) for 30 min at 37 °C. The DNase was inactivated by the addition of 1 μL of RQ1 DNase Stop Solution and incubated at 65 °C for 10 min. The cDNA was generated using a High-Capacity cDNA Reverse Transcription kit (Applied Biosystems, Carlsbad, CA, USA) and oligo d(T)18 primers according to the supplier’s instructions. Reverse transcription was carried out in a 20 μL volume containing 5 μL of RNA extracted from samples or from 10-fold serially diluted virus RNA standard (from 10 to 10^5^ copies) using a PrimeScript RT Kit (Takara) according to the manufacturer's instructions. To test for contamination with transfected DNA, no reverse transcriptase was added into the negative control reaction. The RT-qPCR was carried out on an Mx3005P instrument (Agilent Technologies, Stratagene, La Jolla, CA, USA) using the RealMaster Mix (SYBR Green) Kit (Takara) and primers P23-P26 designed using the VP1 conserved region sequences of CA16 or primers P39-P40 for GAPDH. The RT-qPCR assay was carried out in a 20 μL volume consisting of 9 μL of 2.5 × RealMaster Mix/20 × SYBR Green solution containing HotMaster Taq DNA Polymerase, 1 μL of 5 μmol/L of each oligonucleotide primer and 4 μL of cDNA template. For negative controls, 4 μL of double-distilled H_2_O or 4 μL of template from the no reverse transcriptase reaction was used in plsce of the cDNA template. Amplification of the target fragment was carried out as follows: initial activation of HotMaster Taq DNA Polymerase at 95 °C for 2 min, followed by 45 cycles of 95 °C for 15 s, 57 °C for 15 s and 68 °C for 20 s. The copy number of the target cDNA in the RT-qPCR was determined by using a standard curve of 10-fold serial dilutions of non-linearized plasmid DNA containing the target VP1 sequence (ranging from 10^2^ to 10^9^ copies). Absolute RNA copy numbers were calculated by using standard dilution curves of plasmids containing the target sequence. The sensitivity of the assay or limit of detection was determined to be the lowest copy number that was amplified consistently within the linear portion of the standard curve.

### Western blot

Briefly, equal volumes of supernatants of HEK293T cells transfected with infectious clones (WT or mutant strains) were harvested and boiled in 1X loading buffer (0.08 M Tris, pH 6.8, with 2.0% SDS, 10% glycerol, 0.1 M dithiothreitol and 0.2% bromophenol blue) followed by separation on a 12% polyacrylamide gel. Proteins were transferred onto a PVDF membranes for Western blot analysis. The membranes were incubated with antiserum against CA16 obtained from rabbits immunized with CA16 CC024 or mouse anti-hemagglutinin (anti-HA) mAb (Covance, Princeton, NJ, USA) or mouse anti-tubulin (Abcam, Cambridge, MA, USA) diluted 1:2000 in PBS plus 1% milk, followed by a corresponding alkaline phosphatase (AP)-conjugated secondary antibody (Jackson Immunoresearch, Suffolk, UK) diluted 1:1000. Proteins were visualized using the substrates nitroblue tetrazolium (NBT) and 5-bromo-4-chloro- 3-indolyl phosphate (BCIP) obtained from Sigma (St. Louis, MO, USA).

### Neonatal mouse challenge test

Care and use of animals in the experimental procedures were approved by the Office of Laboratory Animal Management of Jilin University, and the experiments were carried out in accordance with accepted guidelines. One-day-old specific pathogen-free (SPF) ICR neonatal mice (weighing 1.8–2.0 g, provided by the Experimental Animal Center, College of Basic Medicine, Jilin University) were divided randomly into different experimental groups, with three litters per group and 8–10 neonatal mice per litter. The neonatal mice were inoculated intracerebrally with the same dosage of different virus strains or MEM cell culture medium. For infectious clones, the viruses were released by freeze-thawing transfected HEK293T cells and supernatants with WT or various mutant infectious clones. Thereafter, the viruses were cleared by centrifugation at 4500 × *g* for 30 min, passed through a 0.22-μm filter (Millipore, Billerica, MA, USA) and titered in Vero cells. The grade of clinical disease was scored as follows: 0, healthy; 1, lethargy and inactivity; 2, wasting; 3, limb shaking and weakness; 4, hind-limb paralysis; 5, moribund or death. Body weight, activity and the occurrence of limb paralysis, morbidity and death were recorded for 21 days post-infection. The control mice were healthy throughout the experiments.

### Histopathological analysis

Nine mice were sampled: including three dying mice from infected with WT viruses group (with obvious pathological features) and three mice from infected with M2 viruses group and three normal mice from the MEM control group. Various tissue samples from the organs of the infected or non-infected mice, including brain, lung, spinal muscle and hind-limb muscle, were fixed in 10% formalin for 3–5 days. All tissues were dehydrated through an ethanol gradient and then embedded in paraffin before obtaining 4 mm sections for further hematoxylin and eosin staining. Histopathological analysis of the tissues was performed under a light microscope.

### Luciferase assays

HEK293T cells in 12-well plates were transfected with 1 μg 5′UTR-pGL3-Basic (WT or M2) and 50 ng pRenilla for 12 h, 24 h, 36 h and 48 h before harvesting. Luciferase activity was detected in cells using Fluoroskan Ascent FL (Thermo Scientific, Waltham, MA, USA) with the Dual-Luciferase Reporter Assay System (Promega).

### Immunoprecipitation

To identify if the WT or M2 mutant 5′UTR of CA16 can bind to hnRNP K, hnRNP A1 or PCBP1, VR1012, hnRNP K-HA, hnRNP A1-HA or PCBP1-HA expression vector was co-transfected with the WT or M2 mutant 5′UTR expression vector into HEK293T cells. At 48 h after transfection, the cells were harvested and washed twice with cold PBS and then disrupted with lysis buffer (PBS containing 1% Triton X-100, complete protease inhibitor cocktail (Roche, Basel, Switzerland) and RNase inhibitor (New England BioLabs) at 4 °C for 30 min. Cell lysates were clarified by centrifugation at 10,000 × *g* for 30 min at 4 °C. Anti-HA agarose beads (Roche) were mixed with the pre-cleared cell lysates and incubated at 4 °C for 3 h on an end-over-end rocker. The reaction mixtures were then washed six times with cold lysis buffer. Part of each bead pellet was resuspended in 1 × loading buffer for immunoblotting, and another part was resuspended in 1 ml Trizol for RNA extraction according to the manufacturer’s instructions. RNA levels of WT or M2 virus or GAPDH were detected by RT-qPCR with primer P35/P36 and P37/P38.

### Sequences and RNA secondary structure prediction

The full-length genomic sequence of the CA16 CC024 strain was obtained in our previous study^35^. RNA secondary structures were predicted by Mfold^37^ and illustrations were produced with RnaViz^38^.

### Statistical analysis

All data represent at least three independent experiments and are expressed as the mean ± standard deviation (SD). Statistical comparisons between two groups were made using the Student’s *t*-test, whereas comparisons between multiple groups were carried out using one-way ANOVA. *P* values of less than 0.05 were considered to represent a statistically significant difference.

## Additional Information

**How to cite this article**: Li, Z. *et al*. Identification of a nucleotide in 5′ untranslated region contributing to virus replication and virulence of Coxsackievirus A16. *Sci. Rep.* **6**, 20839; doi: 10.1038/srep20839 (2016).

## Figures and Tables

**Table 1:** Primers used in this study.

Primer	Sequence (5′-3′)	Enzyme site	Purpose
P1	TTGCGGCCGCTCCGGAAGCTAATACGACTCACTATAGTTTTAAAACAGCCTGTGGG	*Not* I	CC024(1-2305) amplification
P2	CCCATATGGTTATAATGCCAG	*Nde* I	CC024(1-2305) amplification
P3	CCATATGGTATCAAACTAAC	*Nde* I	CC024(2298-6246) amplification
P4	CGTAGCATGCTTCCTC	*Sph* I	CC024(2298-6246) amplification
P5	GGAGGAAGCATGCTACGG	*Sph* I	CC024(6229-7409) amplification
P6	CGAGCTCTTTTTTTTTTTTTTTTTTTTTTTT	*Sac* I	CC024(6229-7409) amplification
P7	CTGTTTTATAACCCTTTTCCTAAGCAGCAACT	None	M1
P8	AGTTGCTGCTTAGGAAAAGGGTTATAAAACAG	None	M1
P9	CCCTTCCCCTAAGTAGCAACTTAGAAGCTT	None	M2
P10	AAGCTTCTAAGTTGCTACTTAGGGGAAGGG	None	M2
P11	AGCAACTTAGAAGCTCTATACAATCACGACCAA	None	M3
P12	TTGGTCGTGATTGTATAGAGCTTCTAAGTTGCT	None	M3
P13	CAGTCACGTCTTGGCCAAGCACTTCTGTATC	None	M4
P14	GATACAGAAGTGCTTGGCCAAGACGTGACTG	None	M4
P15	GAAAACGTTCGTTATCCGGCTAACTACT	None	M5
P16	AGTAGTTAGCCGGATAACGAACGTTTTC	None	M5
P17	CTAATACAGACATGGTGTGAAGAGTCTATTGAGCT	None	M6
P18	AGCTCAATAGACTCTTCACACCATGTCTGTATTAG	None	M6
P19	CGTGTTTCCTTTTATTTCTTATTGGCTGCTTATG	None	M7
P20	CATAAGCAGCCAATAAGAAATAAAAGGAAACACG	None	M7
P21	GGTGTCTAACAGAGCTGTTGTTTACCTATTTGTT	None	M8
P22	AACAAATAGGTAAACAACAGCTCTGTTAGACACC	None	M8
P23	TCGAGTTAAAACAGCCTGTGGGTT	*Xho*I	5′UTR-pGL3-basic
P24	TTTCCTACAGTTAAGGAGCAATAT	*Hin*dIII	5′UTR-pGL3-basic
P25	GTTAAAACAGCCTGTGGGTT	*Xho*I	5′UTR-pGL3-basic
P26	AGCTTTTCCTACAGTTAAGGAGCAATAT	*Hin*dIII	5′UTR-pGL3-basic
P27	GCGTCGACATGGATGCCGGTGTGAC	SalI	PCBP1
P28	TAGTTTAGCGGCCGCTACGCGTAATCTGGGACGTCGTAAGGGTAGCTGCACCCCATGCC	NotI	PCBP1
P29	GCGTCGACATGTACCCTTACGACGTCCCAGATTACGCGGAAACTGAACAGCCAG	SalI	hnRNPK
P30	ATAGTTTAGCGGCCGCTTAGAAAAACTTTCCAGAATACTGC	NotI	hnRNPK
P31	GCGTCGACATGTCTAAGTCAGAGTCTCCTAAAG	SalI	hnRNPA1
P32	CGGGATCCTTACGCGTAATCTGGGACGTCGTAAGGGTAAAATCTTCTGCCACTGCC	BamHI	hnRNPA1
P33	GCGTCGACTTAAAACAGCCTGTGGGTTG	SalI	5′UTR-VR1012
P34	CGGGATCCTTCCTACAGTTAAGGAGCAAT	BamHI	5′UTR-VR1012
P35	CTACTTCGAGAAACCTAGTAG	None	RT-qPCR CA16-5′UTR
P36	AGCCACTGTCACGGTCGC	None	RT-qPCR CA16-5′UTR
P37	CCCATCACCATCTTCCAGG	None	RT-qPCR GAPDH
P38	TTCTCCATGGTGGTGAAGAC	None	RT-qPCR GAPDH
